# Identifying and describing school-age children who would benefit from AAC: A scoping review of survey tools

**DOI:** 10.4102/sajcd.v72i1.1136

**Published:** 2025-10-25

**Authors:** Bathobile C. Ngcobo, Juan Bornman

**Affiliations:** 1 Centre for Augmentative and Alternative Communication, Faculty of Humanities, University of Pretoria, Pretoria, South Africa

**Keywords:** augmentative and alternative communication, little or no functional speech, scoping review, school-age children, survey

## Abstract

**Background:**

For decades, teaching and learning have relied primarily on oral communication. However, learners with little or no functional speech (LNFS) require augmentative and alternative communication (AAC) strategies to support learning and interaction.

**Objectives:**

This scoping review aims to map available survey instruments used to identify and describe learners who could benefit from AAC, and to highlight research gaps in this area.

**Method:**

The scoping review methodology, guided by the Johanna Briggs Institute was followed. A comprehensive search was conducted across nine databases: Academic Search Complete, Health Source: Science/Academic Edition Nursing, ERIC, Africa Wide Information, Scopus, PubMed, MEDLINE, CINAHL and PsycInfo. The search terms were combined using BOOLEAN operators. Studies were included if they: (1) involved learners aged 5–21 years with LNFS, (2) addressed any educational context, (3) report on any screening instrument (4) were published after 1985, (5) were written in English and (6) presented primary data in a peer-reviewed journal.

**Results:**

Following PRISMA-ScR guidelines, 890 articles were identified, 251 duplicates were removed. Of the remaining 639, 14 underwent full-text review, and seven met the inclusion criteria. No standardised survey instruments were found. Most clinicians adapted or created surveys to meet specific needs, although common variables were assessed, such as learner characteristics and AAC strategies.

**Conclusion:**

The absence of standardized tools to identify learners with LNFS reveals a significant research gap.

**Contribution:**

This review underscores the urgent need for standardised instruments to guide clinical ad educational practices.

## Introduction

Speech-language therapists (SLTs) play a vital role in supporting both receptive and expressive language development across oral and written modalities. Their work spans speech sound correction, oral language skills and phonological awareness, as well as early literacy skills, such as print knowledge, spelling, reading comprehension and fluency – underscoring their essential contribution within mainstream education settings (Stephenson et al., 2024). Children who cannot rely on speech alone to meet their communication needs are routinely placed in schools for learners with special educational needs (LSEN schools) (Department of Education [DoE], 2001). In these settings, the role of SLTs expands to include the assessment, diagnosis and treatment of learners with a range of speech and language impairments resulting from varying conditions (Sharma, 2021). Many learners in LSEN schools would benefit from the implementation of augmentative and alternative communication (AAC) to support either receptive or expressive language. According to Beukelman and Light (2020), AAC employs a range of strategies and tools, including unaided approaches such as manual signs, gestures and finger spelling, as well as aided approaches, which involve tangible objects, line drawings and graphic symbols displayed on communication boards or incorporated into speech-generating devices. As such, implementing AAC strategies can thus support or provide alternatives for learners with impairments in speech and language comprehension and/or expression, encompassing both spoken and written communication. However, learners who could benefit from AAC face significant challenges. The population is highly diverse, making it difficult to accurately identify and describe them. As a result, many of these learners remain ‘hidden’, a situation further exacerbated by the lack of standardised definitions, terminology and prevalence data.

In the international educational and rehabilitation literature, as well as in clinical practice, the terminology includes, but is not limited to: minimally verbal; non-speaking; non-verbal; complex communication needs (CCN); or little or no functional speech (LNFS); or the archaic and misleading ‘mute’. These terms have been operationalised differently. The term ‘minimally verbal’ has been defined as speaking fewer than 20 spontaneous novel words in a 20-min language sample (Almirall et al., 2016), while ‘non-verbal’ refers to a lack of functional communication, including the absence of consistent use of vocalisations, eye gaze and/or gestures to communicate (Franco et al., 2013). The term ‘non-verbal’ shares a similar intent with the earlier label ‘non-speaking’, which described individuals without speech (Glennen & DeCoste, 1997). ‘Non-verbal’ gained popularity as it contrasts with ‘verbal’, a term that, although related to language, is ambiguous, often implying speech in colloquial use (Alant et al., 2006). In this context, ‘non-verbal’ literally means ‘without words’, which could be interpreted as ‘without language’. Additionally, ‘non-verbal’ also implies a lack of speech.

To find a more accurate description of individuals who need or use AAC, the International Society for AAC adopted the term ‘persons with complex communication needs’ in the premier journal within the field of AAC (Alant et al., 2006). While this term was politically correct at the time and carried a strong advocacy orientation, some researchers reported that it was too broad, as it would include conditions like stuttering, which is not the focus of the AAC field. This led to a more precise definition of CCN, specifying that it refers to individuals who produce fewer than 30 intelligible words (Beukelman & Light, 2020). Some authors, however, set the bar at 15 or fewer intelligible words (Cantwell & Baker, 1985). The overly simplistic term ‘mute’ used to describe children who presented with minimal to no language comprehension, but utter non-communicative sounds (Hingtgen & Churchill, 1969), is no longer in use, as it carries negative or outdated connotations.

Notably, the terms described above have been used interchangeably in literature and practice. Ultimately, all these terms refer to individuals who cannot rely on speech to meet their communication needs, thereby representing an expressive communication disability. This highlights the lack of a standard definition or consistent terminology to describe this population – individuals who do not (or cannot) use speech – while also recognising their full range of communication abilities, rather than focusing solely on the absence of speech (Koegel et al., 2020). It is of special note that the Convention on the Rights of Persons with Disabilities (CRPD), an international human rights treaty adopted by the United Nations almost 20 years ago, recognises that communication is a basic human right for all (United Nations, 2006). Organisations such as CommunicationFIRST take this further and encourage the use of descriptive terms and not to use euphemisms such as ‘non-verbal’ and ‘CCN’; as these are factually incorrect (CommunicationFIRST, 2023). This article argues that it is not necessary to quantify the exact number of intelligible words that these individuals can produce. Instead, a qualitative approach is more appropriate, focusing on the fact that their speech is insufficient to meet their daily communication needs. Accordingly, this article will adopt the descriptive term LNFS as used by other scholars (Mophosho & Masuku, 2021; Wendelken & Williams, 2023).

It is imperative to understand that although children with LNFS have a significant communication disability, they form a heterogeneous group. These children can be of any age, from any ethnic or cultural group or any socio-economic background (Beukelman & Light, 2020). They can also experience a range of difficulties with their receptive language skills, which may manifest in difficulties following given instructions, responding to questions and even understanding stories.

Little or no functional speech is further associated with both developmental disabilities (such as autism spectrum disorder) and acquired disabilities (such as traumatic brain injury). Apart from the diagnoses that differ, so might the severity, and hence, their speech may be slightly, obviously or severely unintelligible (Andersen et al., 2010). Therefore, learners with LNFS cannot fully participate in classroom-based activities, as teaching and learning are predominantly presented using verbal communication (Bornman, 2021). Learners with LNFS present with known activity limitations and participation restrictions, of which AAC, if used appropriately, may minimise the barriers experienced (Mophosho & Masuku, 2021). As a result, these children would benefit from AAC system(s) and strategies to either supplement their poor expressive communication skills or, in other cases, provide an alternative to their natural speech (Beukelman & Light, 2020).

In line with international policies (e.g., the Convention on the Rights of the Child, which was ratified in 1995) and local legislation as captured in Chapter 2 of the Bill of Rights and in Section 29 of the *Constitution of South Africa* (*Constitution, 1996*) as well as in the subsequent *South African Schools Act of 1996* (DoE, 2001; Istaryatiningtias & Daliman, 2021), all children between the ages of 7 and 15 – including those with disabilities – have the right to basic education. The limited speech of learners with LNFS typically results in poor classroom participation (Beukelman & Light, 2020; Bornman, 2021) and barriers to learning (both academically and socially). In addition to supporting Beukelman and Light’s (2020) definition of AAC, the Department of Education also highlights that AAC systems and strategies help to alleviate barriers to learning (DoE, 2001), thus making it a reasonable accommodation to ensure educational equity for these children. The DBE (2011) defined reasonable accommodations to include specialised equipment and assistive technology to support LSEN. In line with this, Mophosho and Masuku (2021) described AAC as a human rights tool, not only a receptive and expressive communication tool, but also a means to access education and achieve optimal educational outcomes. Research shows that successful AAC implementation requires teacher training (Mophosho & Masuku, 2021). When trained in AAC strategies, teachers’ perceptions of their own skills, their learners’ communication abilities and classroom inclusion improve (Ngcobo & Bornman, 2024).

One of the greatest challenges in providing services to children with LNFS in educational settings is the lack of descriptive data, which hinders effective service planning. As highlighted earlier, the unavailability of such data is attributable to, among others, the variability and fluidity in the terms used to describe the population who need and/or use AAC. For these learners, the absence of standardised tests or a unified ‘test battery’ that considers both receptive and expressive skill components (Koegel et al., 2020) means that clinicians often rely on informal assessment tools and clinical judgement. This approach is adapted from general language assessment, where SLTs have long developed custom-made screening, identification and even assessment measures (or a combination thereof) (Pascoe et al., 2010). Augmentative and alternative communication assessments are further complicated by the heterogeneity of the population. For example, how an assessment is conducted varies significantly between a child with a physical disability and a child with a cognitive disability, even though both children might need AAC. Furthermore, there are no standard tools for screening or assessing children with LNFS that are linked to specific AAC solutions (i.e., a learner with a specific score on a specific test would benefit from a specific AAC solution). To address this pressing need, this scoping review aims to systematically catalogue existing survey instruments for identifying and describing school-age children with LNFS, who could benefit from AAC. These findings can inform the development of a tool to conduct descriptive studies, providing SLTs with foundational data to guide service planning for this population.

## Research methods and design

### Research design

A scoping review was undertaken to map the survey instruments currently available for identifying and describing school-age children with LNFS who could benefit from AAC, to develop a comprehensive and up-to-date understanding of the topic, and to summarise the existing published data. Scoping reviews attract interest as a research method, as they provide a solid map of existing literature on research topics and unify the identified knowledge (Kazi et al., 2021). Scoping reviews are also evolving as decision-making tools, making them attractive for the current study (Peters et al., 2021). This scoping review used the standardised five-step approach first suggested by Arksey and O’Malley (2005) and updated by the Joanna Briggs Institute (JBI) (Peters et al., 2020), and follows the Preferred Reporting Items for Systematic Reviews and Meta-Analyses – Extension for Scoping Reviews (PRISMA-ScR) framework (Tricco et al., 2016).

#### Step 1: Identifying the research question

To guide the inclusion of literature, the review question was structured using the PCC-format (Population–Concept–Context) as recommended by JBI (Archibald et al., 2016): *What published survey instruments* (Concept) *are available to identify and describe children (5–21 years) with complex communication needs* (Population) *who would benefit from AAC in the school environment* (Context)?

#### Step 2: Identifying the relevant studies: Search strategy and study selection

A librarian experienced in systematic review methodology evaluated the search strategy and suggested relevant education, health sciences and humanities databases. A systematic search of nine relevant databases (Academic Search Complete, Health Source: Science/Academic Edition Nursing, Eric EbscoHost, Africa Wide Information, Scopus, PubMed, MEDLINE EbscoHost, CINAHL, APA PsycInfo) was conducted to capture potential articles. This ensured methodological rigour and alignment with the broader landscape of review types, including scoping reviews. Research has shown that involving a librarian in the review process results in more accurate search methods and a higher quality review (Pawliuk et al., 2024). Search terms using keywords were generated through the PCC method, as shown in Table 1. The keywords included Boolean operators AND and OR to link the PCC components in the search, and truncation (*) expanded the search.

**TABLE 1:** Search terms in population-concept-context format table.

**P** (patient/ population/problem)	‘school age child*’ OR ‘elementary school child*’ OR ‘learners’ OR ‘primary school child*’ OR youth AND ‘complex communication needs’ OR CCN OR ‘non-verbal communication’ OR ‘communication impairment’ OR ‘communication disability’ OR ‘speech disability’ OR ‘language disability’ OR ‘little or no functional speech’ OR LNFS OR ‘augmentative and alternative communication’ OR ‘AAC’
**C** (Context)	‘special needs education’ OR ‘special education’ OR ‘remedial education’
**C** (Concept)	‘screening tools’ OR ‘survey’ OR ‘questionnaire*’

**FIGURE 1:**
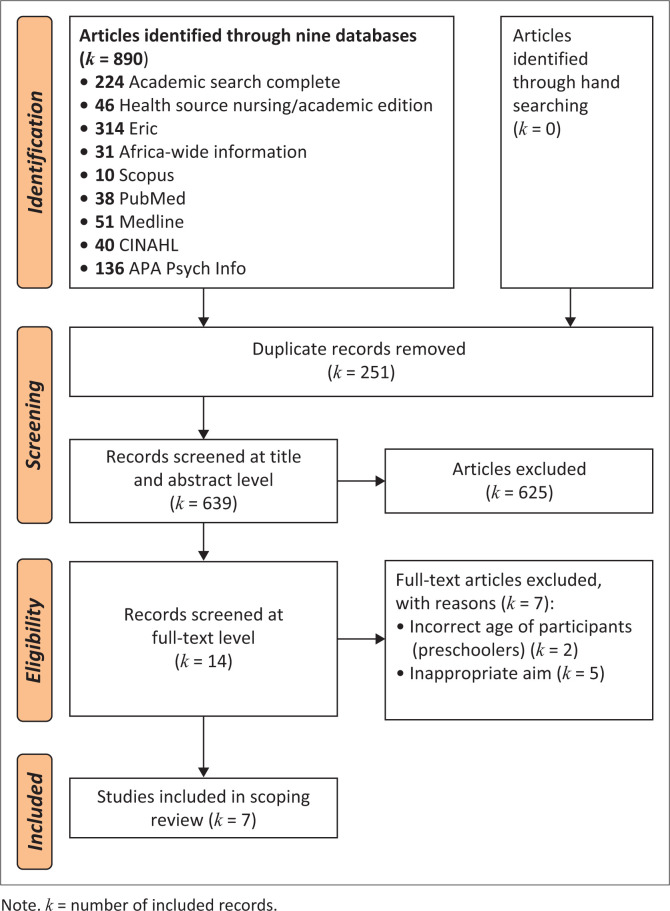
Preferred reporting items for systematic reviews and meta-analyses – ScR flow diagram outlining the details of the search strategy.

The 890 records identified namely Academic Search Complete (*k* = 224), Health Source Nursing (*k* = 46), Eric (*k* = 314), Africa Wide Web (*k* = 31), Scopus (*k* = 10), PubMed (*k* = 38), MEDLINE (*k* = 51), CINAHL (*k* = 40), APA PsycInfo (*k* = 136). The complete process is shown in Figure 1, using the PRISMA format.

#### Step 3: Selecting the included studies

The screening process was guided by the following seven eligibility criteria: (1) only studies that reported on children with LNFS between the ages of 5 and 21 years old; (2) conducted in an educational setting; (3) reported on a screening tool or instrument; (4) published between January 1985 and February 2025. The start date was set as 1985, marking the publication of the International Society for AAC’s first AAC journal (McNaughton & Light, 2015); (5) reporting on primary data; thus, all scoping or systematic reviews and meta-analyses were excluded; (6) including all study designs to widen the scope as much as possible and (7) because of limitations in time, human and financial resources, only studies published in English were included (Dobrescu et al., 2021).

The 890 records identified included a total of 251 duplicates removed. The remaining 639 records were imported into Rayyan, a digital platform that supports working together on scoping reviews, as Rayyan automates the screening and selection of relevant study processes, thereby saving researchers valuable time (Ouzzani et al., 2016). The use of the Rayyan platform enhanced inter-rater agreement and contributed to a more objective selection of studies (Johnson & Phillips, 2018). The 639 records, uploaded onto Rayyan, were screened on the title and abstract level, of which 625 were excluded for not aligning with the focus of the current scoping review.

The screening at the title and abstract level was performed by two independent reviewers, reaching a 97.9% inter-rater agreement. The 13 records that were in conflict were discussed with a third independent reviewer, with full consensus from all reviewers. The inclusion and exclusion criteria were used to exclude studies at the abstract level.

After screening the 639 records at an abstract level, 14 records remained, of which two reviewers independently read the full texts and evaluated them for eligibility. Seven of the 14 records were excluded after full-text review, mostly as the aim was incorrect (i.e., focusing on comparing learners who use AAC to those who do not). The inter-rater agreement was 100%, which is regarded as a high agreement (Tricco et al., 2016). The results from Step 3, the selection of the included studies, are shown in Figure 1, using the PRISMA-ScR diagram.

#### Step 4: Charting the data

To ensure consistent and independent data reporting, a data extraction tool was developed. General information was included, such as population, the context and the concept. Similar to the screening, two reviewers independently extracted the data, and any disagreements were resolved through consensus meetings, leading to the data being uploaded to a Microsoft Excel spreadsheet.

#### Step 5: Collating, summarising and reporting the results

The extracted data were synthesised to address the research question and provide an overview of the breadth of the literature on the topic. The results of the included studies were collated and categorised according to specific topics that emerged during data extraction. As this scoping review included only seven articles, the data are presented qualitatively, using tables to illustrate the key themes and findings, while also alluding to gaps in the existing research, which can guide future studies.

### Ethical considerations

An application for full ethical approval was made to the Research Ethics Committee, University of Pretoria, Faculty of Humanities, and ethics consent was received on 17 February 2025. The ethics approval number is 20806762 (HUM019/1024).

## Results

The results of the scoping review are presented as descriptive information related to the seven included studies (e.g., author(s), year of publication, the main aim of the study and the country), the population (e.g., age, number and sex of the participants) and the study context (e.g., participant description and place of data collection) (see Table 2). An analysis of the concept (i.e., the survey instrument) described in the various studies then follows (see Table 3).

**TABLE 2:** Descriptive information of each study included.

Descriptive information on study						Participant information				Context
#	Author(s)	Year	Main aim of the study	Study design	Region / country	Participant age / grade	Number of participants	Participant sex	Participant description	Place of data collection
1	Burd et al.	1988	To describe data on non-verbal school-age children in North Dakota	Survey	North Dakota, US	5 – 21 years	279 persons 17 schools, hospitals, and residential institutions (completed by agencies)	57% males 43% females	Non-verbal: speaking less than 15 words	Special school (i.e., schools for learners with: intellectual disability, deaf, blind) Hospital Residential institution for handicapped children Evaluation centre
2	Bornman	1995	To describe children’s communication and related behaviours as perceived by their classroom teachers to assess the need for communication intervention (with special reference to AAC) within this context	Survey	Pretoria, South Africa	3–12 years	412 learners 55 teachers	66.3% males 33.7% females	Non-speaking: speaking less than 15 words	Special schools for learners with intellectual disability
3	Weiss et al.	2005	To determine the number of children and adolescents with CCN in special education preschools/schools in Israel To gather information about the learner’s educational programmes, especially about AAC systems, equipment, usage and intervention strategies	Survey	Haifa, Israel	3–21 years	5430 learners 118 SLTs, OT, School principals	Not disclosed	Severe communication impairment	Special education preschools Schools
4	Stehr	2008	To determine the prevalence of children with LNFS enrolled in special schools for children with severe intellectual impairment in Cape Town To describe their communication skills To describe their exposure to AAC intervention at school	Survey	Cape Town, South Africa	6–15 years	190 learners 18 teachers	Not disclosed	LNFS; non-speaking; speaking fewer than 30 intelligible words	Special schools
5	Andzik et al.	2018	To describe the characteristics of learners with communication needs as reported by their special education teachers To describe their reported level of proficiency in communication To describe the presence of challenging behaviour among these learners To describe the type and amount of AAC training of the special education teachers supporting these learners	Online survey	50 states US	4–22 years	15 643 learners 4031 special education teachers	Not specified	CCN	Special schools
6	Binger et al.	2021	To provide prevalence and caseload estimates for learners with highly unintelligible speech in New Mexico To describe the demographic makeup of this population To describe basic educational and AAC provision for this population	Online survey	New Mexico, US	Grade K-12 (Approx. 5–18 years)	1668 learners 323 caseloads (completed by SLTs)	68% males 32% females	Learners with highly unintelligible speech; no more than 50% intelligible; Learners who communicate non-verbally	Special schools
7	Coan-Brill et al.	2025	To provide descriptive data on the factors affecting children’s need for, access to, and use of AAC systems To develop a profile of Canadian children with cerebral palsy and speech and motor limitations who require AAC, and to improve AAC use.	Online survey	Western Ontario, Canada	18 months – 18 years	21 children 21 caregivers 39 clinicians	57% males 43% females	Speech limitations	Rehabilitation service centres (i.e., preschools, special schools, hospitals, etc.)

Note: Please see full reference list of this article: Ngcobo, B.C., & Bornman, J. (2025). Identifying and describing school-age children who would benefit from AAC: A scoping review of survey tools. *South African Journal of Communication Disorders, 72*(1), a1136. https://doi.org/10.4102/sajcd.v72i1.1136 for more information.

LNFS, little or no functional speech; SLTs, speech-language therapists; CCN, complex communication needs; Approx., approximately; AAC, augmentative and alternative communication; OT, occupational therapy; US, United States.

**TABLE 3:** Concept investigated related to the learners.

Concept										
#	Survey type	Number of questions	Survey duration	Informants	Question format (e.g., biographical, medical, communication)	AAC investigated	Learner academic performance and skills investigated	Prevalence rate	Response rate	Recommendations from the study
1	Article-based survey	Not specified	Unspecified	Special education personnel agencies	***Learner variables:*** Biographic data Grade levels in academic areas Type of educational placement (e.g., regular class, resource room) Resource services (e.g., speech therapy [ST], occupational therapy [OT]) History of verbal skills AAC confirmation	Sign language Handwritten language Typewritten language Pointing communication board Electronic communication device	Reading Math Written language Handwriting Spelling Adaptive behaviour (i.e., self-help)	15.25% per 10 000	100%	Further research to define the population The definition used of children who speak 15 words or less is precise for future studies Identify the types of services required by these children
2	Article-based survey	22	12 min	Teachers	***Learner variables:*** Biographic data Comprehension skills Communication skills Services received (e.g., ST, OT) AAC confirmation	Speech & vocalisations Head nodding Gesture (e.g., pointing) Facial expressions Sign language Finger spelling Communication board with objects, pictures or symbols Typing/writing	Reading Writing Math	38.3%	100%	Replicate study in 10 years: ■ To determine changes in prevalence ■ To determine the use and availability of AAC systems
3	Online survey	18	Unspecified	Speech-Language Therapists Occupational Therapists School principals	***Learner variables:*** Biographic data AAC: confirmation of use and systems listed	Natural gestures Sign language Communication board with graphic symbols Speech-generating device Switch-activated toys	None mentioned	40.8%	64.5%	Identify the prevalence of children with CCN who attend schools where Hebrew is not spoken Identify CCN among adults with medical conditions Determine the use and availability of AAC systems Determine prevalence of learners who use AAC to influence staffing and case loads Document professionals’ and caregivers’ AAC knowledge
4	Article-based survey	12	12 min – 20 min	Teachers	***Learner variables:*** Biographic data Communication skills Services received (e.g., ST, OT) AAC confirmation	Speech Sounds Head nodding Gesture (e.g., pointing) Facial expressions, Sign language Finger spelling Communication board with: objects, pictures or symbols Typing/writing	Reading Writing	31.05%	100%	Determine teachers’ perceptions of children with LNFS’ receptive language abilities Replicate study in diverse SA contexts in urban and semi-rural schools, to inform policymakers and educational planners on AAC service delivery
5	Online survey	6-question loop	Unspecified	Special education teachers	***Learner variables:*** Disability Primary mode of communication (i.e., speech, gestures, etc.) IEP goals Challenging behaviours	Speech Gesture Pictures Speech-generating devices	None mentioned	Gesture – 6.9% Pictures – 6.5% SGD – 4.8%	Not calculated	Provide more detailed demographics of learners with communication impairments Use random sampling measures Calculate response rate Describe the teacher’s level of AAC training
6	One online survey with a series of stages, with a comments section	19	5 min – 10 min	Speech-Language Therapists	***Learner variables:*** Biographic data (e.g., disability, gender) Indicate the number of learners with highly unintelligible speech AAC: confirmation of use, systems listed Therapy received	Natural gestures (e.g., head nodding) Sign language Object symbols Communication boards with pictures and graphic symbols Low tech High tech	None mentioned	1 in 89 learners (1.12%)	65%	Replicate study in other states to strengthen results Develop clear definitions to ensure accurate description of all learners who require AAC
7	Online survey	Questions not specified (three classification instruments used)	Not specified	Caregivers Speech-Language Therapists Occupational Therapists Physiotherapists	***Learner variables:*** Biographic data – age only AAC: confirmation of need, accessand use	Facial expressions Speech sounds Gestures (e.g., pointing) Manual signs Eye gaze Low tech High tech (e.g., partner-assisted scanning, switches	None mentioned	38% who use AAC 31% who need AAC	Not given	Determine the rates of need, access and use of AAC among all children with cerebral palsy in Canada

#, number; LNFS, little or no functional speech; SLTs, speech-language therapists; CCN, complex communication needs; AAC, augmentative and alternative communication; IEP, individual educational plan; SGD, speech-generating device.

### Descriptive information on included studies

Despite the importance of descriptive studies and the fact that the number of non-speaking school-age children is unknown, few studies have been undertaken. The descriptive information shown in Table 2 clearly demonstrates that most of the seven included studies were conducted in the United States (US) (#1, #5, #6), with two studies conducted in South Africa (#2, #4), one study each in both Canada (#7) and Israel (#3). One study was conducted in the 1980s (#1), one was conducted in the 1990s (#2), two studies were conducted in the 2000s (#3 and #4), one study was conducted in 2018 (#5) and two studies were conducted between 2021 and 2025 (#6 and #7).

### Population

Table 2 shows that in four studies (#1, #2, #6 and #7), more males were reported than females, while the other three studies (#3, #4 and #5) did not report their participants’ gender. This study’s primary context was LSEN; however, the inclusion criteria allowed for a combination of settings, of which an LSEN school must be part. Having mentioned that, study #7 included children from 18 months old; the children’s ages mostly ranged from 3 (#2, #3) to 22 years old (#5). Study #6 described learners according to their grades (K-12), which translates to approximately 5–18 years of age. Table 2 also shows that the sample sizes of the seven included studies ranged considerably, from 21 learners (#7) to 15 643 (#5).

### Context

Table 2 shows that learners who could benefit from AAC were described using different terms in the various studies; for example, the term CCN was used in the US study (#5), ‘severe communication impairment’, was used in the Israeli study (#3) and ‘speech limitations’ in the Canadian study (#7). Studies #1 and #2 aimed to quantify the number of words that the learners could intelligibly speak, by describing the learners as ‘speaking less than 15 intelligible words’. Study #4, from South Africa, used the term LNFS, which was quantified to mean ‘less than 30 intelligible spoken words’. Study #5 also attempted to quantify the percentage of words spoken intelligibly as ‘less than 50% intelligible words’. All studies collected their data in either special schools (#2, #4, #5, #6) or combined sectors, such as hospitals, preschools, rehabilitation centres and residential homes (#1, #3, #5).

### Concept

The review revealed that all studies made use of custom-made surveys. In terms of their distribution and presentation format, three were article-based (#1, #2, #4) and four were online (#3, #5, #6, #7). Table 3 shows that survey completion times ranged from 5 min to 20 min (#6, #2, #4), while studies #1, #3, #5 and #7 did not specify the completion time. One South African study (#2) included the highest number of questions, being 22, closely followed by a US study (#6) with 19 and the Israeli study (#3) with 18. The other South African study (#4) and US study (#5) included 12 and six looped questions, respectively. A combination of informants was noted; three studies used teachers (#2, #4, #5), one study used SLTs only (#6) and two studies used a combination of clinicians (#3, #7). Interestingly, study #3 also used principals, whereas study # 7 also used caregivers as informants. Study #1 used special education personnel agencies only.

A similar pattern was noted regarding the question format of the studies. All studies, excluding studies #5 and #7, presented a similar pattern in terms of investigating the learner variables in terms of the biographic information. Study #5 only wanted the disability label and # 7 only wanted the age. A similar pattern was noted in terms of the response formats. A combination of responses was noted with the survey instruments, such as multiple-choice, rating scales, binary (yes/no) questions and open-ended text fields (#3, #5, #2, #7). The studies also presented a similar pattern in terms of listing the different AAC systems that were considered, including both unaided (e.g., speech sounds and natural gestures) and aided systems (e.g., communication boards with objects and electronic communication devices). The Israeli study (#3) and two US studies (#5, #6) did not include any factors related to school performance or skills of learners, while both South African studies (#2, #4) and one US study (#1) investigated the school performance of learners, skills and functionality.

Five of the seven studies reported a response rate of higher than 60%, while one study (#5) did not calculate the response rate, and another (#7) did not report the response rate. Table 3 also shows the main outcomes in terms of prevalence rates of the different studies. The prevalence rate varied greatly: in the two South African studies (#2, #4) and the Israeli study (#3), a prevalence rate of more than 30% was reported, while the US studies (#1, #6) presented a prevalence rate below 2%. The one US study (#5) and the Canadian study (#7) presented their prevalence in terms of the percentage of AAC used by learners with LNFS.

### Future recommendations from studies

All studies presented similar recommendations. Studies #1, #5, #6 and #7 recommended that a robust and unambiguous definition of the AAC user population should be developed, as it is essential to ensure accurate identification and description of individuals who require AAC support. Such clarity would facilitate more precise needs analyses, inform appropriate AAC device provision and support effective allocation of staff and resources, ultimately contributing to more equitable and efficient service delivery. Studies #2, #3 and #4 all recommend that in future studies, AAC service provision, AAC services and AAC use should also be described in greater detail for this population to influence policymakers and educational planners.

## Discussion

Speech-language therapists play a vital role in assessing and managing learners with LNFS in special schools (Sharma, 2021). The results from this scoping review highlight the limited availability of instruments that can be used to identify and describe learners with LNFS. Only seven studies were identified, with three originating from the US (#1, #5, #6), two from South Africa (#2, #4) and one each from Israel (#3) and Canada (#7). This scarcity may be because of several factors, including ongoing challenges in clearly defining the population of interest and selecting the most suitable methodologies for data collection. The literature reflects variation in how this population is conceptualised. Some authors focus on providing descriptive accounts (e.g., #3, #5, #7), while others attempt to quantify specific characteristics associated with the group (e.g., #1, #2, #4, #6). For instance, Sutherland et al. (2005) employed the term CCN to characterise the population more broadly. In contrast, South African studies defined participants more specifically by quantifying expressive language abilities, referring to learners as using fewer than 15 intelligible words or fewer than 30 intelligible words (Alant, 1999; Bornman & Alant, 1996). This poses a serious research challenge because of the lack of uniformity in terminology, which could lead to miscommunication, confusion and a misinterpretation of research findings (Smoktunowicz et al., 2020).

In addition to differences in how the population was defined, the studies also varied in the level of biographical detail reported. Some studies did not disclose the gender of participants (Sutherland et al., 2005), while others provided both gender information and male:female ratios (Erickson & Geist, 2016; Lillehaug et al., 2023). Moreover, there was considerable variation in the ages of participants across the included studies. Some studies reported on children as young as 18 months (e.g., #7), while most studies focused on school-aged children, with a few also including individuals up to school-leaving age (e.g., 20 years old). As noted by Heidari and colleagues (2016), researchers who omit detailed biographical information may do so because they do not perceive it as essential to their study objectives. The lack of agreement on biographical characteristics and definitional boundaries across the available studies precludes the possibility of conducting a meaningful comparison across studies. Standardisation in these areas is essential to ensure data compatibility and interpretability across different research contexts.

In addition, the findings also indicate that most of the studies presented a similar pattern in terms of investigating the learner variables when attempting to identify and describe learners who could benefit from AAC. Typically, a list of the different AAC systems, including both unaided and aided systems, was provided (Alant, 1999; Binger & Light, 2006; Erickson & Geist, 2016; Sutherland et al., 2005). No pattern was observed in terms of investigating the school performance of learners, skills and functionality, with some studies including these factors (Alant, 1999), while others did not (Sutherland et al., 2005).

Furthermore, there is no set protocol on who to use as informants for gathering descriptive data. The findings indicate that in high-income countries, the informants included agencies (#7), school principals (#3) and clinicians (SLTs) (#6). In contrast, in the low- and middle-income country (LMIC) included in the study – South Africa – teachers were used as informants (#2, #4). The rationale being that in the absence of SLTs, teachers were deemed well-positioned to identify and describe learners with communication difficulties, as well as describe learners’ functional performance.

Linked to the variation in informants is the challenge of determining appropriate data collection settings. In all the studies included in the review, data were collected either in LSEN schools (#2, #3, #4, #5, #6) or across a combination of sectors such as hospitals, preschools, rehabilitation centres and residential homes (#1, #7). These settings may have been selected based on the age range of the children studied. However, collecting data primarily in schools presents a significant limitation, as it may exclude children who are not currently enrolled in educational settings. The reasons for non-enrolment may vary; however, such children could still benefit from AAC interventions (Zhang & Holden, 2025).

Another aspect linked to who provided the information is related to the best method of collecting data. Similar to the findings noted in the review, some of the earlier descriptive studies were article-based (Alant, 1999; Bornman & Alant, 1996; Sutherland et al., 2005), while some of the later studies were online-based (Binger & Light, 2006; Erickson & Geist, 2016). In LMICs, article-based surveys are preferred over online surveys because of various factors such as limited Internet access, digital literacy, population coverage, data collection infrastructure and cultural and social factors.

The findings also indicated a variation in the time needed to complete the surveys. Some studies did not specify the time needed to complete their survey (Sutherland et al., 2005), while others were described as ‘short’ (e.g., Alant, 1999; Bornman & Alant, 1996). The literature consistently agrees that surveys should be concise; subsequently, lengthy instruments and questions can lead to respondent fatigue and incomplete responses (Ghafourifard, 2024). The findings from this review suggest that there is no set guidance in reporting response rates. Some studies presented a high response rate (#1, #2, #3, #4, #6); in contrast, some did not calculate the response rate (#5), which poses a challenge when wanting to understand the limitations of the given findings (McMillan & Schumacher, 2010).

While some insight into learners with LNFS has been gained, there remains a critical need for more robust and geographically diverse research. A key challenge is the lack of consensus on terminology, which hinders both the identification and description of this population and limits comparability across studies. In addition, decisions regarding which biographical and contextual variables to include, such as age, gender, diagnosis and educational placement, are often inconsistent, limiting the generalisability of findings. Methodological variations further compound these issues, as studies differ in their selection of participants, data collection strategies and the materials used. Establishing greater standardisation in definitions and methodologies would enhance the comparability of research outcomes and ultimately contribute to a more accurate and comprehensive understanding of individuals with LNFS, which could in turn positively impact service delivery.

### Limitations

This review yielded valuable information; however, some studies on AAC as a specific intervention for identifying and describing learners with LNFS may have been inadvertently excluded because of the applied framework and eligibility criteria. Furthermore, the search strategy was limited to studies reporting on primary data, a specific time period and the English language.

### Recommendations for future studies

There is a need to develop a standardised tool – led or co-developed by SLTs – to accurately identify and describe learners with LNFS, and determine the most appropriate AAC systems that would benefit these learners.

## Conclusion

Surveys used to identify and describe learners with LNFS who could benefit from AAC present with little to no uniformity. There is no standard uniformity in terms of the survey structure or even the data to be collected. Surveys are typically custom-designed to align with specific research objectives. However, biographical information and details about AAC systems learners use or require are common elements found across most of these surveys. Given their expertise in communication development and intervention, SLTs play a critical role in both designing and administering these surveys, as well as in interpreting the data to inform individualised AAC recommendations and support strategies.
